# Exploring Fit in a Mobile Health Intervention for Alcohol Use Disorder: Qualitative Study

**DOI:** 10.2196/65218

**Published:** 2025-10-14

**Authors:** Nora Jacobson, Linda S Park, Alice Pulvermacher, Samantha Voelker, Mallory Herzog, Andrew Quanbeck

**Affiliations:** 1 Institute for Clinical and Translational Research, School of Medicine and Public Health, University of Wisconsin-Madison, Madison, WI, United States; 2 School of Nursing, University of Wisconsin-Madison, Madison, WI, United States; 3 Department of Family Medicine and Community Health, School of Medicine and Public Health, University of Wisconsin-Madison, 610 N Whitney Way, STE 200, Madison, WI, 53705-2700, United States, 1 608-262-7385

**Keywords:** alcohol use disorder, EPIS framework, fit, mHealth, mHealth app, effectiveness, implementation trial, qualitative study, motivations, opinions, drinking behavior

## Abstract

**Background:**

Implementation frameworks such as the Exploration, Preparation, Implementation, Sustainment model emphasize the importance of the *fit* between an intervention and its context, which includes the needs of its target population, as well as the culture, resources, and capabilities of the implementing organization. Although lack of fit is a major barrier to implementation, fit has not often been a focus of implementation research. This paper uses fit as a lens to examine the implementation of Tula, a mobile health app aimed at reducing risky drinking days among individuals meeting the criteria for mild to moderate alcohol use disorder, in a 3-arm (app alone, app plus peer mentoring, and app plus health coaching) randomized controlled trial.

**Objective:**

We sought to better understand the trial results and to provide actionable guidance for future implementation of digital health interventions in health care organizations.

**Methods:**

Semistructured interviews with 18 trial participants and 7 Tula implementers were conducted. Trial participants were pulled equally from each arm of the trial and represented participants who demonstrated both high and low engagement with the app. Implementers consisted of a project manager, 4 peer mentors, and 2 health coaches. Interviews with participants focused on their motivations, opinions, and experiences of the intervention and their perception of their drinking behavior following the intervention, including how their use of the app worked to change that behavior. Interviews with implementers were centered on their roles, theories of change, perceptions of intervention, and areas for improvement. All interviews were analyzed using rapid qualitative analysis with deductive and inductive components.

**Results:**

We identified areas of both fit and misfit. For example, there was a good fit between implementers’ theories of change and participants’ description of how change occurred. Fit was improved by the versatility of the app, which allowed participants to customize their experiences. Conversely, misfit was noted in the app’s inability to cultivate connection for many participants and a disjunction between the role of peer mentors in the intervention and their broader professional ethos.

**Conclusions:**

Focusing on fit provides a useful guide to enhance future iterations of the Tula app that lead to better sustainment of the intervention.

## Introduction

Excessive alcohol use is linked to increased injury risk and poor health outcomes, causing approximately 140,000 US deaths and 3.6 million years of potential life lost annually [1,2]. Alcohol use disorder (AUD) spans a spectrum of risk, necessitating interventions tailored to an individual’s likelihood of developing severe AUD [3]. Despite its high prevalence and significant impact on health, alcohol use is often undertreated in general medical settings. Many patients with AUD go unrecognized or do not receive appropriate intervention due to time constraints, limited training, and stigma associated with addressing substance use in primary care [4].

Health coaching is increasingly used in health care organizations as a strategy to promote behavior change, support chronic disease management, and enhance patient engagement. Its integration into clinical care is growing, particularly within primary care and population health initiatives [5]. Peer support models are increasingly integrated into health care systems, particularly within mental health services, to enhance recovery-oriented care and patient engagement. This integration reflects a global shift toward incorporating lived experiences into treatment frameworks to improve outcomes and support system transformation [6].

Improving access to effective interventions is crucial due to AUD’s widespread and destructive nature [1,7]. Digital medicine apps show promise in treating and providing ongoing care for patients with AUD, offering a widely accessible means for self-management and clinical monitoring [8]. However, research on implementing these technologies for early intervention in AUD is limited [3]. A key research question remains: how can human support enhance the effectiveness of digital medicine apps [9-11]?

This paper reports results from the qualitative component of a hybrid effectiveness–implementation trial that systematically varied the degree of human touch offered to support use of an evidence-based mobile health (mHealth) intervention for AUD (NCT04011644) [12]. Implementation of the intervention was guided by the EPIS (Exploration, Preparation, Implementation, Sustainment) framework, which delineates 4 phases in the implementation of evidence-based interventions: *exploration* to consider target population needs, *preparation* to assess barriers and facilitators to implementation, *implementation* to assess progress and determine adjustments, and *sustainment* to assess how to maintain the context structures and supports of the intervention [12]. For successful implementation, there needs to be a high degree of “fit” between the intervention and the contexts of implementation [13,14]. Fit, which has also been described as “appropriateness” and “compatibility” [14-16], has been conceptualized as encompassing multiple domains, including alignment between the intervention and the needs and preferences of the target population and alignment between the intervention and the culture, resources, and capabilities of the implementing organization [14]. The lack of fit is one of the most often cited barriers to implementing an intervention, but the construct has not often been the focus of implementation research [14].

In this paper, we examined the experiences of the users and implementers of an mHealth intervention called Tula (Sanskrit for “balance”) for AUD that was tested in a recent 12-month randomized controlled trial [17,18]. We deploy the notion of fit as a lens to explore the alignment between the Tula intervention, the various digital support models, the target population, and the implementing organizations to better understand the trial results and to provide actionable guidance for future implementation of digital health interventions in health care organizations.

## Methods

### Intervention Design

The Tula intervention used a harm reduction approach to support individuals with mild to moderate AUD to decrease the number of heavy drinking days (defined as days with 5 or more standard drink units for males and 4 or more for females). Trial volunteers were randomized to 1 of 3 arms that included different levels of human touch: app only, app plus peer support, or app plus health coaching (see Table 1 for details of these arms). Participants in each arm received a monetary incentive to complete periodic study surveys that assessed the number of heavy drinking days and several quality of life measures. A full description of the protocol can be found in a study by Park et al [17].

Full trial results are reported in a study by Quanbeck et al [18]. Across all 3 groups, a statistically significant time effect was observed, with the percentage of self-reported heavy drinking days dropping from 38.4% (95% CI 35.8%-41%) at baseline to 22.5% (95% CI 19.5%-25.5%) at 12 months. There were no significant differences between groups in alcohol use reduction. Participants in the health coaching arm showed statistically significant improvements in mental health–related quality of life compared to the other 2 groups. However, participants in the health coaching arm dropped out of the study at a higher rate than participants in the other 2 arms, making that result difficult to verify due to risk of bias due to survivorship effects (ie, patients who had worse mental health may have been more likely to withdraw from the study).

**Table 1.** Study arms.

	App only	App+peer support	App+health coaching
Use of Tula	Unguided use of Tula	Tula use supported by a community-based peer mentor	Tula use supported by the health coach
Intervention	Study team conducts safety monitoring and technical support	Interpersonal communication and wellness monitoring via the app	Up to three 1:1 health coaching sessions via phone call
Discussion forum	No discussion forum	A discussion forum moderated by a peer mentor	A discussion forum moderated by a health coach
Communication	No communication feature with private messaging	The private messaging feature in communication routing to a peer mentor	The private messaging feature in communication routing to a health coach
Dashboard	No dashboard access	No dashboard access	Health monitoring by health coaches supported by a dashboard

### Participant and Implementor Description

Eligibility criteria are listed in Textbox 1. All participants created anonymous usernames to use the app.

We partnered with a community-based organization to have certified peer specialists provide social support for participants randomly assigned to the peer support group. We provided project-specific training regarding the app and facilitating discussions. Peer specialists were not assigned to any specific participant but were available to any participant who reached out to them via a private messaging feature of the app. Peer specialists were able to draw upon their lived experiences with recovery from substance use disorders, including mental health issues, to moderate a discussion forum while encouraging participants to use the app.

In total, 2 certified health coaches—part of the health care system—worked one-on-one with participants randomly assigned to their group. The health coaches’ goal was to help participants achieve their self-identified goals toward a healthier lifestyle as they reduced their alcohol consumption. Participants were encouraged to use up to three 1:1 personalized health coaching sessions via phone during the first 90 days of their trial session.

Textbox 1. **Eligibility criteria.**

**Inclusion criteria**

- 21 years old or older
- Wants to reduce drinking
- Owns a smartphone and is willing to download and use the Tula app
- Lives within the health care system service area
- Meets at least one of the following criteria:
  · AUDIT (The Alcohol Use Disorders Identification Test) [19] screening score 8+, OR
  · Responds “yes” to at least 2 questions on the Alcohol Use Disorder: Diagnostic and Statistical Manual of Mental Disorders, 5th Edition [AUD: DSM-5] [20], OR
  · Reports moderate- to high-risk drinking patterns
     · 4+ drinks on any single day and >7 drinks per wk (women and men aged >65 y)
     · 5+ drinks on any single day and >14 drinks per wk (men)

**Exclusion criteria**

- Reports symptoms consistent with severe alcohol use disorder during screening (6 or more of 11 Diagnostic and Statistical Manual of Mental Disorders, 5th Edition [DSM-5] criteria)
- Active psychotic disorder diagnosis
- Acute medical problem requiring immediate hospitalization
- Terminal illness   

### App Design

**Figure 1.**
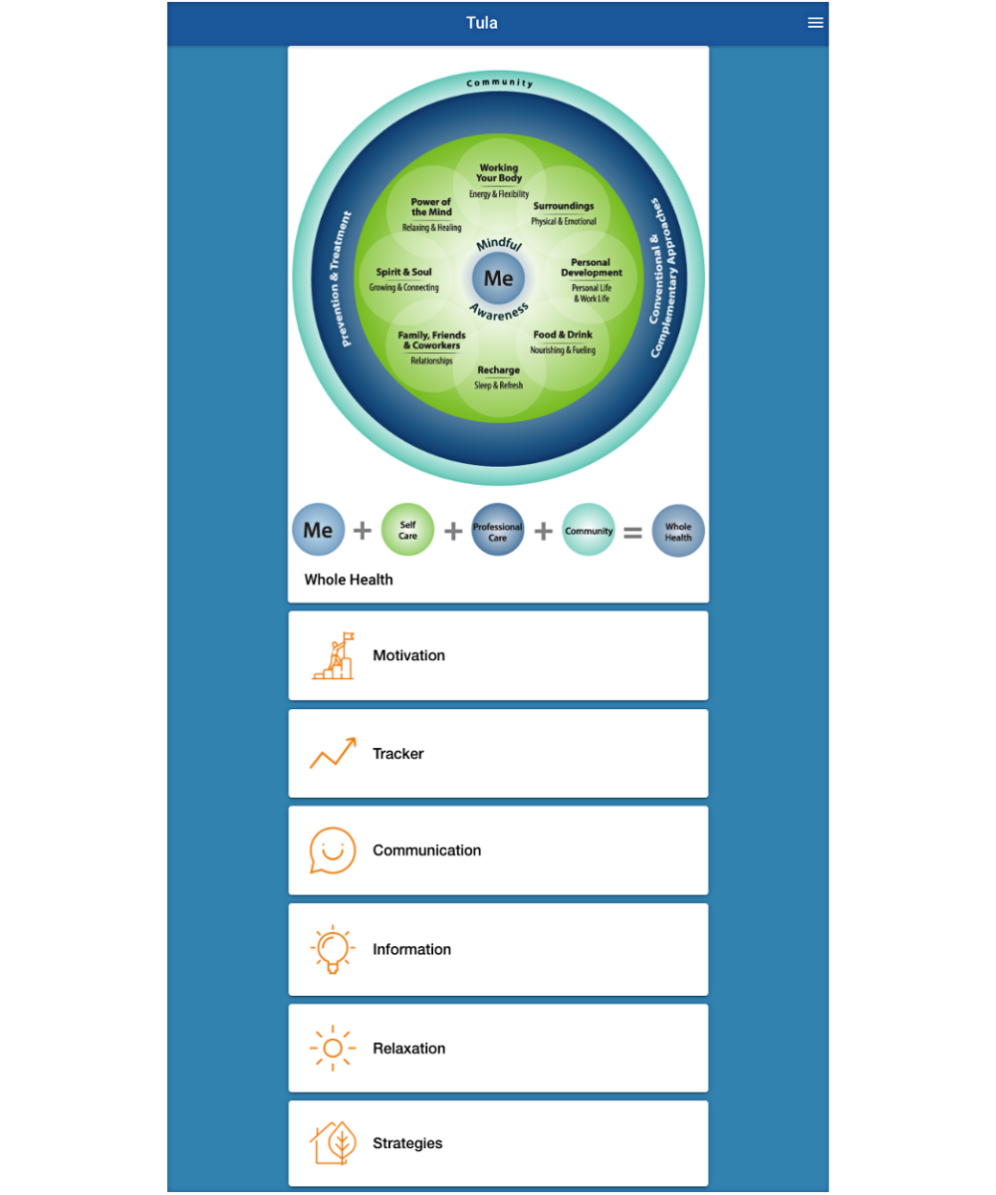
View of the app.

The app was designed according to the tenets of self-determination theory, which posits that adaptive functioning is optimized when people are able to meet their needs for autonomy, competence, and connection [17,21]. The app content was informed by the Whole Health Model, an approach to health promotion that views health as having physical, psychological, spiritual, and social dimensions and seeks to enable people to improve their health by empowering them to identify their own needs and priorities and set their own goals [22]. Figure 1 shows a visual depiction of the app.

The original trial protocol for this Tula intervention project was considered minimal risk for participants and was exempt from requiring a formal data monitoring committee review. All data collected via the app were stored in secure, password-protected servers. Participants were informed that no patient health information would be collected from their medical records, nor would any study data be entered into their medical records. All data were deidentified before any analysis.

### Qualitative Study Sampling

We sought to elicit perspectives from implementers of the Tula intervention and research participants. The implementer sample (n=7) included 1 research team member who had project management responsibilities, all 4 individuals who worked as peer mentors, and the 2 individuals who provided health coaching. We used the level of engagement with the Tula intervention as a criterion to selectively sample research participants. Engagement was determined by total number of app page views over 12 months for participants in all 3 arms, discussion group views and posts for the participants in the peer support arm, and number of health coaching sessions completed for those in the health coaching arm. Participants were divided into high and low engagement groups (with the most engaged listed first in their group and the lowest engaged listed first in theirs). Researchers then used these rankings to invite interview participation, with the most and least engaged invited first. All invitations were sent via email addresses provided by the research participants. Research staff attempted contact with each participant 3 times before moving on to the next participant on the list. Participants who did not engage at all—for example, those in the health coaching arm who attended zero coaching sessions—were not eligible because they would have no direct experience with the intervention.

### Qualitative Interviews

Implementers were interviewed by the first author, a public health scientist and qualitative methodologist. These interviews focused on roles played in Tula, implementers’ theories of change, perceptions of how the intervention did or did not work, and suggestions for improvement of the intervention. Trial participant interviews were conducted by the first author and 2 other research team members, AP, a research program manager, and SV, an undergraduate research assistant, who participated in interview training conducted by the first author. The 3 interviewers, all women, were not involved in developing the Tula intervention and had not had previous contact with the participants. Participant interviews covered participants’ reasons for joining the study and their opinions of and experiences with the different components of the intervention, as well as their perceptions of whether and how Tula had affected their drinking behavior. Interviews were conducted using semistructured interview guides developed by the research team and minimally revised following early interviews. All interviews were held virtually and included only the interviewer and the research participant. Interviewers did not have knowledge of the individual participants’ outcomes—that is, whether or not they had reduced their drinking.

### Qualitative Analysis

All interviews were audio recorded, transcribed verbatim, and analyzed using rapid qualitative analysis, an approach that includes both deductive and inductive elements and is particularly appropriate for implementation research [23]. An initial coding matrix used domains derived from the EPIS framework: innovation, inner context, and outer context. After sorting the data into these a priori categories, a round of coding developed new categories that were specific to the data set. For example, the inner context included participants’ motivations for volunteering for the study, whereas the outer context included the impact of the COVID-19 pandemic (which coincided with the trial). The notion of fit as described by Blanchard and Livet [14] was then operationalized by searching for areas of compatibility and incompatibility between the Tula intervention and the inner and outer contexts of its implementation. Other analytic activities included memo writing and discussions among the research team about the developing findings. The first author led the analysis, including an iterative process of review and feedback from the other authors.

### Ethical Considerations

The full trial was approved by the Minimal Risk Institutional Review Board (IRB) at the University of Wisconsin-Madison (2019‐0337 CP012 for IRB Study 2019‐0337, NCT06305624). In addition, the trial received a Certificate of Confidentiality from the National Institutes of Health. The qualitative study was approved by the same IRB as an addendum to the trial protocol (2019‐0337 CP012 for IRB Study 2019‐0337). The IRB allowed a waiver of written consent for the trial, but all participants were provided with a study information and consent document and were given the opportunity to ask any questions about the study at the point of recruitment. Similarly, interview participants were provided with written information about the purpose of the interviews and their rights as research participants. Verbal consent was affirmed at the beginning of each interview. Interviews were anonymized during transcription, which was completed by a University of Wisconsin–based service certified in Health Insurance Portability and Accountability Act (HIPAA) regulations and human subjects protections. All research participants interviewed received US $50 gift cards in appreciation for their time. Implementer interview participants were not compensated apart from the financial support they received for their roles in the intervention.

## Results

### Overview

The findings reported here were based on semistructured interviews with 7 Tula implementers and 18 research participants. All implementers approached agreed to take part in interviews. The participant response rate varied considerably, from a low of 15% in the low engagement app only arm to a high of 75% in the high engagement app only arm. The average response rate across arms and engagement levels was 41.25%. Nonresponders included participants whose email addresses proved to be nonfunctioning, as well as those who never replied to 3 invitations. Implementer interviews ranged from 30 to 70 minutes in length. Participant interviews ranged from 19 to 55 minutes (mean 27.5 min). The demographics and usage data for the 18 research participants were largely reflective of the entire trial sample (n=558). Interview participants were primarily White (17/18, 94.4%), female (13/18, 72.2%), and highly educated, holding a bachelor’s or master’s degree (12/18, 66.7%). The average age was 47 (SD 12.8) years (see Table 2, which also shows use data for interview participants and the overall trial sample).

**Table 2.** Characteristics of the study population^a^.

Characteristics	Low use (n=9)	High use (n=9)	Total interviewed (n=18)	Total (n=558)
Age, mean (SD)	43.8 (13.9)	50.2 (11.6)	47 (12.8)	42.8 (12.9)
Female, n (%)	6 (66.7)	7 (77.8)	13 (72.2)	366 (65.6)
Race, n (%)				
White	9 (100)	8 (88.9)	17 (94.4)	510 (91.4)
Black	0 (0)	1 (11.1)	1 (5.6)	27 (4.8)
AI/NA^b^	0 (0)	0 (0)	0 (0)	5 (0.9)
Asian	0 (0)	0 (0)	0 (0)	12 (2.2)
NH/PI^c^	0 (0)	0 (0)	0 (0)	2 (0.4)
Other	0 (0)	0 (0)	0 (0)	6 (1.1)
Hispanic or Latino, n (%)	0 (0)	0 (0)	0 (0)	14 (2.5)
Education, n (%)				
<High school	0 (0)	0 (0)	0 (0)	3 (0.5)
HS^d^ or GED^e^	0 (0)	1 (5.6)	1 (5.6)	67 (12)
Vocation or associate	2 (22.2)	3 (33.3)	5 (27.8)	85 (15.2)
Bachelors	5 (55.6)	5 (55.6)	10 (55.6)	235 (42.1)
Masters	2 (22.2)	0 (0)	2 (11.1)	121 (21.7)
Doctorate	0 (0)	0 (0)	0 (0)	47 (8.4)
Marital status, n (%)				
Married	6 (66.7)	4 (44.4)	10 (55.6)	301 (53.9)
Widowed	1 (11.1)	1 (11.1)	1 (5.6)	10 (1.8)
Divorced	0 (0)	1 (11.1)	1 (5.6)	55 (9.9)
Separated	0 (0)	0 (0)	0 (0)	6 (1.1)
Never married	0 (0)	2 (22.2)	3 (16.7)	109 (19.5)
Living with a partner	2 (22.2)	1 (11.1)	3 (16.7)	73 (13.1)
Refused	0 (0)	0 (0)	0 (0)	1 (0.2)
Don’t know	0 (0)	0 (0)	0 (0)	3 (0.5)
Severity: mild, n (%)	4 (44.4)	3 (33.3)	7 (38.9)	356 (63.8)
Tula usage, mean (SD)				
Page views	385.8 (119.9)	2587 (1104)	1487 (1365)	806.1 (510.5)
DG^f^ Read	21.5 (26.1)	320.2 (464.2)	170.8 (349.5)	76.7 (118.1)
DG Post	8 (0)	64 (42.5)	50 (44.6)	8.3 (12.1)
Health coach visits	1 (0)	3 (0)	2 (1.1)	1.7 (1.3)

a Mild severity was determined by a screening survey based on National Institute on Alcohol Abuse and Alcoholism thresholds and the Diagnostic and Statistical Manual of Mental Disorders, 5th Edition, when 2 to 3 symptoms/criteria are present for AUD. A total of 4 to 5 symptoms constitute moderate AUD. Six or more symptoms constitute severe AUD—patients scoring 6 or higher were not eligible for the trial.

b AI/NA: American Indian/Native American.

c NH/PI: Native Hawaiian/Pacific Islander.

d HS: high school.

e GED: General Educational Development.

f DG: discussion group.

### Motivations

Research participants identified 2 alcohol-specific motivations for wanting to join the study: being curious about their drinking habits and wanting to cut down on their drinking. In discussing these motivations, they alluded to feelings of being at risk for problem drinking either because of their individual circumstances—for example, a family history of alcoholism or an increase in drinking during the early days of the COVID pandemic—or because they perceived their social context as being oriented toward heavy drinking.

Why? I guess I was just kind of, I was curious about my own habits. Alcoholism runs in my family...and I just was kind of, it’s always kind of lingered in the back of my mind, like how responsible I am with alcohol. So I was just kind of curious to see how often I really did drink...and cravings and, you know, did I need it?

Participant 04 in the app-only arm

[I already have anxiety issues, but during COVID my anxiety really went up a lot, and I found that I was drinking quite a bit, like every day actually. Much more than I had before. So when the study came about...I just thought it would be really good to try to get back on track and to decrease the, how much I was drinking, because it really affects my anxiety.]

Participant 06 in the health coaching arm

Participants also described being motivated to participate in the Tula intervention by a wish to improve their overall health and by an interest in research.

[Well, for myself, it’s, you know, like some people have every year where, you know, they start the new year and they’re like, okay, what do I want to try to do to be better? I’m always trying to identify things of, areas work where I can, you know, get better. When I had seen that, I knew that I was probably drinking a little bit more than what I should have, and it just looked at, you know, right time, right place, right opportunity.]

Participant 04 in the health coaching arm

[Well I just wanted to cut down on my drinking, and the reason I wanted to do that was because my cholesterol levels were kind of high. Well, I attributed that to late night snacking, and you know, just overeating because that’s what alcohol kind of triggers in me. So, yeah. So that’s why I wanted to participate, and just to be healthier, you know?]

Participant 06 in the peer support arm

[So I found it very interesting. I have a health background, and I’ve always been, you know, into health and wellness and fitness. So I just thought that it sounded like a very interesting study to be a part of. I’m always kind of interested in research studies in general, but this one in particular I thought was especially interesting just because it had, you know, it related more towards overall, you know, health and wellbeing.]

Participant 01 in the health coaching arm

### Implementer Theories of Change

Each of the 3 components of the Tula intervention—the app, the peer support, and the health coaching—had its own theory of change.

As noted, the app’s design was based on self-determination theory and the Whole Health model. The app’s embedded theory of change held that people’s ability to change their drinking behavior would be promoted by features that were customizable and educational and that supported relationships with others.

Peer mentors told us that behavior changes, such as drinking reduction, depend on an individual’s intrinsic motivation. Motivation is prompted and reinforced by increased self-awareness of the pros and cons of the behavior and, ultimately, a decision that the cons of continuing the behavior outweigh the pros. The role of the peer mentor is to use their own experiences of substance use, recovery, and relapse to establish rapport and then to “support and love [people] where they are at” by offering nonjudgmental, highly personalized, and often intensive assistance.

The health coaches described grounding their practice in the Whole Health model and the Stages of Change model of health behavior change [24]. Similar to peers, they saw health behavior change as originating in an individual’s intrinsic motivation and the coaches’ role as “support[ing] people in making health change that they were ready and wanting to make.” Health coaches “[get] to the heart of what really matters to people” in order to provide tools that facilitate an individual’s “mindful awareness” of their health habits and the ways in which these habits are or are not consonant with what the individual values. Health coaching consists of fairly structured conversations in which coaches “help [people] go from knowing to doing,” or move from contemplation to action in the stages of change, by working with the individual to identify what they value, figure out how their current habits do or do not serve these values, set goals for change, review their progress toward meeting these goals, and fine-tune their goals as needed.

### Change Mechanisms

Participants described 4 main mechanisms through which the Tula intervention worked to support change: accountability, encouragement, information, and connection. These mechanisms were described by participants in each of the 3 arms, though, as we explore, some components of the Tula intervention were a better fit than others in supporting these mechanisms.

Accountability came in 2 forms. In the first form, the Tula intervention raised participants’ awareness of their drinking behavior by providing visibility onto the number of drinks taken each week.

[[The app asks you] “How much did you drink last week?” And that, for me, was enough to just kind of keep me in check. You know, like, “Ooh, I had a lot last week”...if you have three or four drinks, then you want to have a fifth drink. You’re like, “Ooh, but I gotta enter that in that app”.]

Participant 02 in the health coaching arm

[Well, as you know, the application itself makes you more mindful of how much alcohol you’re consuming because on a weekly basis, it asks you to tally up how much you’ve had, and when you actually physically do that, when at the time might seem like I just had a few beers, you know, just had a few drinks, you can see where it really starts to add up. [Laughs] And so I used it, I guess on a weekly basis and then because I was doing that on a weekly basis and seeing how much I was drinking a night... it made me more mindful day-to-day as to how much I was consuming. And so it was, it became more like, “Oh, you know, you already had a couple, you know, maybe that third one is not a good idea.”]

Participant 04 in the peer support arm

In its second form, accountability consisted of cycles of pattern analysis and goal setting and either positive or negative reinforcement for behavior change.

[I also liked that it had the like, mood and sleep like trackers along with the alcohol consumption trackers. Just because it was interesting to kind of see the correlation between all three of those factors and not just looking only at alcohol consumption. It’s kind of interesting to see. I noticed that like alcohol consumption tend to be a little higher, like in weeks where I rated mood or lower sleep.]

Participant 02 in the app-only arm

[[The combination of the tracker and the surveys] made me reflect on my goals... If the question was, how connected do you feel to people outside of work, or, you know, to your family. If I noticed that I connected low there, and then had like three or four drinks above my goal for that week...then that was like a pretty clear, oh, you know, things are out of balance here.]

Participant 02 in the peer support arm

Both high and low engagement participants described accountability as having had the most impact on their drinking behavior.

Encouragement worked by promoting positive thinking, changes in perspective, mood elevation, and hope, which in turn helped to provide motivation to change behavior.

[Oh, just you know, [the thought of the day] was always something positive and it just launches your day in a great manner and, you know, inspiring quotes or, you know, you’ve got this, you can do it. You know, tough times don’t last, tough people do kind of things and just very inspiring, you know, and it wasn’t preachy or anything like that. It was always just kind of nice little encouraging snippets.]

Participant 04 in the peer support arm

[I dug into the gratitude list. So it was more of a reminder. If I was having a bad day that I could really point out, remember, positives of my day to go back and reflect upon.]

Participant 05 in the health coaching arm

[[The peer mentor] kind of opened up and told us about her experiences as well. I thought she was very down to earth, and very encouraging.]

Participant 06 in the peer support arm

[There was challenges and stuff like that and it wasn’t always easy, that there was always being progress made. So that, it was breaking that stuff down when it’s like, oh, well, you know, and [the health coach]'d be like, no, well, you know, look what you’ve already done and, you know, and then come with some other tools or things where I was having difficulties that I wasn’t able to utilize, showing me the way to, you know. Just different ideas and things like that. So it was like, I just felt like I was going in the right direction, and that would be probably the biggest, it was very motivating, I guess.]

Participant 04 in the health coaching arm

Information supported change by offering new strategies for adaptive behaviors, as well as substitutions for maladaptive behaviors.

[[The app contained] just different ideas of things to do to occupy yourself other ways and meditation ideas, like this all kinds of different ideas. Things to keep you busy.]

Participant 05 in the peer support arm

Connection allowed users to exchange practical knowledge and to gain insight into others’ experiences.

[It was insightful to get some tips from other people and it kind of led me to something new I found out, too, which has really helped me. I, well I started making mocktails, and I’d have them at home. I rarely drink at home, but every once in a while, I get really stressed out, beat up, and just, ugh, you know? I want a cocktail, but I found that mocktails were really good for me because they kind of gave me that ritual. They really helped. So, somebody suggested trying them and, you know, try some different recipes, and yeah. So I got some good information from other members that way.]

Participant 06 in the peer support arm

[So it was useful in the fact that...challenges that I would run into, I could see others that were challenged in the same manner, and some, on some occasions you could get feedback of what they were able to use or what they did that that made that difficulty something that was easier to deal with. Then there was also ones that would have comments where you like, okay, I don’t want to do that. So on the positive there was there was good things, and on the negative there was good things. It was just nice to have that different perspective.]

Participant 04 in the health coaching arm

### Fit and Misfit

We found areas of fit and misfit across the 3 components of the Tula intervention and the contexts of implementation.

Tula was a good fit with participants’ motivations for joining the trial. All interview participants described the intervention as having been successful in giving them some insight into their drinking. Many, though not all, reported that they had reduced their drinking or otherwise changed their drinking behavior. Several noticed improvements in their overall health—for example, weight loss or improved sleep.

There was compatibility between implementers’ theories of change and participants’ reports of how Tula worked to effect changes in their drinking behavior. The accountability, encouragement, information, and connection mechanisms were consistent with the theories of change embedded in the 3 components of Tula. That is, when it worked, Tula appeared to have worked in ways it was theorized it would.

There was also a good fit between participants’ motivations, the change processes they described, and the app’s features. The feature most often associated with both forms of accountability was the weekly drink tracker, which was universally popular with low and high engagement participants. All interview participants described using the tracker to become more aware of their drinking behavior, and several attributed their ability to reduce or change their drinking behavior to the tracker. Participants who experienced the second form of accountability also cited the study surveys. The app features described as being the most encouraging were the thought of the day, the modules devoted to motivation and gratitude, and, for those in the human touch arms, interactions with the peer mentors and health coaches. The Tula components that seemed most closely linked to information were the app modules focused on relaxation and drinking reduction strategies and the discussion groups, particularly the group associated with the peer support component. The Tula components mentioned as important to fostering connection were the peer support and the health coaching.

All the peers and health coaches indicated that the harm reduction philosophy underlying Tula was compatible with their own beliefs and approaches. The health coaches described their Tula work as very similar in approach to their non-Tula coaching and as personally fulfilling.

A significant misfit was the dissonance between the peer mentors’ conceptualization of the peer role and the role as conceived in the Tula intervention. Specifically, the peer mentors contrasted the highly personal nature of their services as offered in the non-Tula context with the distanced and anonymous quality of the Tula work, which one peer mentor described as “dry.” All peers noted this difference and suggested that it had hindered their effectiveness.

[I didn’t have enough in-depth dialogue with each participant where I could understand where they’re coming from...in a more in-depth way. And...I couldn’t really share my experiences as deeply as I would if it was face-to-face.]

Peer mentor 01

[There was no face-to-face contact...there was not much of a connection made there. Whereas, you know, with [traditional] peer support, we meet in person, face-to-face. You know, we can, you know, we can see the, the body language...that’s how you build a rapport, connection and trust. And so through an app there just wasn’t anything like that... It’s just not enough. It was, it was just really almost non-human interaction in a way.]

Peer mentor 03

One peer mentor experienced this misfit so acutely that they provided their contact information to trial participants and held one-on-one telephone conversations.

The discussion feature, included in the app for those trial participants randomized to the peer support or the health coaching arms, was successful for some users, especially to the extent that it was a source of information and encouragement. However, it appeared to be largely a misfit when it came to providing deeper feelings of connection. Both the health coaches and the peer mentors said they felt poorly equipped to facilitate meaningful discussions. Even the highest engagement participants in the peer support component, those who spent the most time on the discussion pages, were lukewarm about the discussions. Several participants said they had other outlets, such as AA, that met their needs for connection. Others noted that they felt they had little in common with participants who posted in the discussions.

[I only really read. I never really posted, to be honest... It’s easier just to kind of read other peoples’.]

Participant 03 in the peer support arm

[To be honest, I did not play on the social side of it at all...it was people who weren’t going to quit, and they didn’t really want to. They were looking for someone to talk them into it. That’s kind of the way I looked at those comments...in my opinion, if you want to do something, you just got to do it. It’s not like somebody should be talking to you or anything like that. It’s got to come from inside.]

Participant 01 in the peer support arm

Implementers identified a misfit attributable to the research context. They perceived that some trial participants were motivated not by an authentic wish to change their behavior but by the monetary compensation offered by the study. Thus, they lacked “skin in the game,” and, while these participants completed the surveys that were the basis for receiving compensation, they did not really engage with any of the Tula components.

The app’s technology and the volume of its content showed fit and misfit. While most implementers and participants praised how well the app functioned—“everything you click on, it works,” others described it as clunky and unintuitive or reported technical glitches that interfered with use. In total, 2 participants expressed a lack of enthusiasm for any app: one because their life during COVID was taking place almost entirely online and they wanted a break from screens and another because they were trying to reduce their phone usage.

Many participants liked how much information the app contained; others felt overwhelmed or wanted a more curated experience.

[And so for me it was frustrating because there was so much in abundance. And I felt like you had to do it all. It wasn’t like I could do a subset and be successful. So then I found myself not being as successful. That’s a terrible thing to say, but I did...it became overwhelming and I felt more guilty and worse off.]

Participant 03 in the health coaching arm

Other domains that mixed fit and misfit were preferences related to intervention intensity and privacy.

While most participants seemed to appreciate that the Tula experience was fairly relaxed, others felt that they would have gotten greater benefit from a more intensive intervention.

[I probably would have benefited from it all. You know, having the group, having the counselor and the app, all three of those, would be a pretty high success rate I think, for myself personally.]

Participant 03 in the app-only arm

[I felt [the health coaching sessions] weren’t memorable. I felt they were short, weren’t in-depth. It was like, “what do you want your goals to be? Here are your problems. OK, that’s a good goal.” I just fell off the map. It really wasn’t, I thought it would be more invasive at that level with the coach, and it was 15 minutes maybe each... I felt the coach didn’t coach... You want someone who is skilled but is also going to provide you with expertise and solutions and ideas. I didn’t feel that was done.]

Participant 05 in the health coaching arm

Many participants stated that they did not care to reveal themselves or make themselves “vulnerable” to strangers online, and thus, they appreciated having the ability to remain anonymous.

[Wanting to kind of self-improve on your own terms was really helpful for me in just having it be private, but knowing that, you know, there were strategies in there I could look at and I didn’t have to, like reach out to somebody. For that, personally, for me, it was helpful.]

Participant 01 in the app-only arm

[And that way when you use the app, it’s private, you know? No matter what you enter into it, it’s just you and your entering info, you know? No one else around and it can, as bad as it may seem, who cares? Because no one sees it but you, you know what I mean? It goes to data for whatever you guys are doing, but at the time when you’re entering stuff it don’t matter, because it’s just you.]

Participant 05 in the app-only arm

The fact that health coaching required some self-revelation during one-on-one conversations with coaches was uncomfortable for some participants in that arm.

[The other most difficult thing is talking to the coach on the phone whom I didn’t have a relationship with, didn’t know. And for me, it was already an embarrassing topic…I felt very probed and very kind of like a scientific experiment…I know it was anxiety producing for me.]

Participant 03 in the health coaching arm

Notably, the research team member who informed participants of which arm they had been assigned to reported that many people randomized to the coaching arm expressed disappointment with their assignments. As noted earlier, the health coaching arm had a higher rate of attrition than the other 2 arms.

## Discussion

### Principal Findings

While most of the literature on alcohol reduction mHealth interventions has been inconclusive, there is some evidence of promise across a range of studies [25-28]. Our examination of fit suggests some explanations for that promise.

We found a good fit between the Tula intervention, inclusive of the app, peer support, and health coaching, and many aspects of the contexts of implementation. There was a compatibility between the implementers’ theories of change and participants’ descriptions of how change occurred and between these change processes and several of the app’s features. The best fit was between the change process participants called accountability and the app’s tracking feature. This is consistent with findings from other studies, which have demonstrated positive behavior change from logging alcohol consumption as participants see real-time progress via the tracking function [26]. Giroux et al [29] conducted a study of a location-based monitoring and intervention system for AUDs, and Osth et al [30] compared 2 different smartphone apps to complement alcohol treatment. Both reported that participants found the accountability function and tracking feature increased their awareness of drinking consumption and increased their motivation for behavior change.

We also uncovered several areas of misfit. There was discordance between the peer mentor role in the Tula intervention and key features of peer mentorship in the non-Tula environment. The app’s discussion feature was only minimally effective at fostering connection.

In addition, we observed several domains of mixed fit and misfit, specifically pertaining to the app technology’s ease of use and its content and to participants’ preferences related to intervention intensity and privacy. Patient concerns over data privacy and security are important when it comes to issues of choice in digital health. Patients expect clear guidance on how their data will be used and value the ability to control data sharing [31]. That the Tula intervention offered an array of options—“something for everyone”—was cited by both participants and implementers as an important strength. Having choices meant that participants could customize their experiences, including the choice to ignore parts of the intervention that they did not find helpful or interesting. A narrative review of 71 articles on barriers to and facilitators of digital health adoption found that patient empowerment, self-management, and personalization were the key drivers in the adoption of digital health tools [32].

Another key finding was the ways in which the research context affected both participants’ and implementers’ experiences. For some participants, random assignment constrained choice, pushing them into interventions they had no interest in exploring. From the implementers’ perspective, the incentives offered to trial participants appealed to motivations that had nothing to do with drinking or health. In both cases, the misfit between the intervention and the research context appeared to have threatened Tula’s effectiveness. Conversely, the survey that participants were asked to fill out as part of the research seemed to have functioned as an effective part of the intervention for some participants by reinforcing accountability.

This qualitative examination helps provide an explanation for the main results of the Tula trial. The dominant mechanism of change described by the interview participants was accountability, which was closely tied to the app’s tracker, a feature that trial participants in all three arms were able to access. The fact that the peer support component was not more effective than the app alone may be because what happened in the Tula intervention bore little resemblance to the highly personalized practices of outreach and response that are part of the ethos of peer support as it has developed as a profession [33,34]. The higher rate of attrition in the health coaching group compared to the other study arms may be at least in part explained by a misfit between the privacy preferences of a subset of participants and the demands of the health coaching intervention. However, it should be kept in mind that related research on other digital health apps for alcohol reduction has also shown very high attrition rates; Attwood et al’s examination of the Drinkaware app for alcohol use reduction observed high attrition, with only 42.6% of users using the app after the first week and only 5% retained after 12 weeks [35]. The improvement in mental health–related quality of life among participants who stuck with health coaching seems to reinforce the idea that allowing people to customize their own intervention experiences may garner the best outcomes.

### Limitations

We refer readers to a report of the primary quantitative analysis for a discussion of limitations of the parent trial [18]. The design of this qualitative study did not allow us to determine the exact nature of the relationship between fit and engagement for individuals in the primary quantitative analysis, but the data do suggest that the two are related. Participants for whom the Tula components to which they were assigned were a good fit were enthusiastic about them and appear to have been more engaged. Lower engagement users seem to have experienced more misfit.

Although the demographics of the interview participants were largely consistent with the overall Tula sample, individuals who agreed to participate in interviews might have differed from the larger sample in ways we did not measure. Our decision not to recruit participants who did not use the Tula components to which they were assigned means that the sample does not represent the group of participants with the lowest levels of engagement. However, the consistency of responses suggests that we did achieve informational redundancy and thus are able to represent the experience of participants who met our inclusion criteria. Because the overall sample for the trial was predominantly female, White, and highly educated, the findings reported here may not be generalizable to a more diverse population. Improvements in mental health–related quality of life may have been related to the personalized social support shown in the coaching group, although we do not have specific quantitative data to substantiate this claim. Finally, it could be that participants were interested in reducing the number of heavy drinking days, hence their participation in the study, and that the reduction seen was a result of their own personal motivations—not the app, peer support, or health coaching.

### Future Directions

Further analysis of the specific intervention components that participants engaged with may help improve the intervention design in future research. The study found that different users have different preferences for intervention intensity. Dynamic adjustment of intervention intensity could feasibly be achieved in future intervention designs to meet individual needs by using sequential, multiple assignment randomized trial designs or microrandomized trial designs [36,37]. Future research should also explore the impact of comorbid factors common in patients with AUD (eg, depression, anxiety) on the fitness and effectiveness of mHealth interventions.

### Conclusions

The results reported in this paper suggest that the concept of fit can go some way toward explaining the outcomes of the Tula trial and may provide insights into participant experiences of mHealth interventions in general. Using fit as a lens through which to examine these experiences provides insights that can be applied to iterative intervention improvement, which in turn will increase effectiveness and promote sustainment.

## Abbreviations

AUD: alcohol use disorder
IRB: institutional review board
mHealth: mobile health
